# Fertility in young-onset colorectal patients with cancer: a review

**DOI:** 10.1093/oncolo/oyae141

**Published:** 2024-06-21

**Authors:** Qiuping Jiang, Hongmei Hua

**Affiliations:** Department of Nursing, The Second Affiliated Hospital of Zhejiang University School of MedicineHangzhou, Zhejiang, People’s Republic of China; Department of Nursing, The Second Affiliated Hospital of Zhejiang University School of MedicineHangzhou, Zhejiang, People’s Republic of China

**Keywords:** colorectal cancer, young-onset colorectal cancer, fertility preservation, oncofertility, oncological treatment

## Abstract

Although the overall incidence and mortality of colorectal cancer have declined, diagnosed cases of young-onset colorectal cancer have increased significantly. Concerns about future fertility are second only to concerns about survival and may significantly affect the quality of life of young cancer survivors. Fertility preservation is an important issue in young-onset colorectal patients with cancer undergoing oncotherapy. Here, we discussed the effects of different treatments on fertility, common options for fertility preservation, factors affecting fertility preservation and improvement measures, and the relationship between fertility and pregnancy outcomes in young-onset colorectal patients with cancer.

## Teaser

Fertility preservation is an important issue in patients with young-onset colorectal cancer. This article reports the effects of different treatments on fertility, common options for fertility preservation, factors affecting fertility preservation and improvement measures, and the relationship between fertility and pregnancy outcomes in this patient population.

**Implications for practice**

The incidence of young-onset colorectal cancer has significantly increased. Treatment of young-onset colorectal cancer requires specific attention to fertility-related issues, including the impact of cancer treatment on fertility, factors affecting fertility, fertility preservation, and the relationship between fertility and pregnancy outcomes. We recommend that healthcare professionals pay attention to the fertility issues of patients with young-onset colorectal cancer, and in cancer treatment, it is recommended that patients undergo multidisciplinary discussions, including reproductive health.

## Introduction

Although population-based screening has reduced the overall incidence of colorectal cancer (CRC), there has been an astonishing increase in young-onset colorectal cancer (YO-CRC, defined as a diagnosis of CRC before age 50).^1^ Evidence shows that by 2030, one-tenth of colon cancer and a quarter of rectal cancer will be diagnosed in people under 50 years.^2^ With the trend of younger patients with CRC and delayed childbearing, some patients have not yet had children or intend to have children again. Studies indicate that 70% of young patients have high reproductive needs.^3^ However, due to the contradiction between the damage to fertility caused by cancer treatment and the high reproductive needs of patients,^4^ as well as patients’ concerns about children’s health,^5^ young females with CRC are prone to high levels of reproductive problems, which will affect their quality of life and treatment compliance. Therefore, the American Society of Clinical Oncology (ASCO) and the American Society for Reproductive Medicine (ASRM) recommend that healthcare providers discuss the possibility of infertility and options for fertility preservation with all patients of reproductive age diagnosed with cancer.^6,7^

However, in a previous study on male patients with cancer, only 29% of these patients received fertility counseling, and 11% attempted sperm banking.^8^ Another study showed that although 60% of oncologists reported being aware of the ASCO’s fertility preservation guidelines, less than 25% of respondents regularly followed these guidelines, distributed educational materials, or referred patients for fertility preservation discussions.^9^ Unresolved fertility issues may significantly affect the quality of life of cancer survivors of childbearing age. Therefore, understanding the reasons for the impact of cancer treatment on fertility, analyzing the obstacles that affect fertility, and proposing improvement measures can reduce the regret of cancer survivors, improve their physical and mental health, and enhance their quality of life.

This review briefly summarized the impact of treatment on fertility, reviewed common fertility preservation options, analyzed the influencing factors of fertility protection and proposed improvement measures, and explored the relationship between fertility and pregnancy outcomes in YO-CRC patients. Although the focus was on patients with YO-CRC, some of the information may also apply to any cancer patient who is about to receive gonadotoxic treatment.

## Effect of treatment for YO-CRC on fertility

Compared with late-onset CRC, YO-CRC is characterized by aggressive, more advanced, and systemic treatment.^10^ Young patients were more likely to receive adjuvant or neoadjuvant therapy, surgery, and multiagent systemic therapy.^11,12^ Fertility may be affected to varying degrees by a single surgical treatment, or combined chemotherapy or radiotherapy.

Surgical resection is the main treatment for localized early CRC. Studies have shown that low anterior resection (LAR) related to rectal cancer can affect the sexual function and fertility of patients.^13^ Machačkova et al evaluated the sexual function outcomes of patients after LAR using the Female Sexual Function Index (FSFI) and the International Index of Erectile Function questionnaire (IIEF). The results showed that the IIEF and FSFI were significantly lower at 6 months after ileostomy closure.^14^ A retrospective study found that women with ulcerative colitis (UC) who did not undergo ileal pouch-anal anastomosis (IPAA) had a higher birth rate (46.8 children/1000 years) than those who underwent IPAA (27.6 children/1000 years).^15^ Surgery can also damage the bladder and sexual function of rectal cancer patients, mainly due to the division of the pelvic autonomic nerves during surgery.^16^ Male neurological damage may affect ejaculation, and if the disease is locally advanced, it may involve the removal of female reproductive organs. In addition to neurological damage that may affect sexual function, the presence of a stoma is also associated with poorer sexual function.^17,18^

Radiation therapy can lead to male infertility,^19^ premature ovarian failure, or infertility in women.^17,20^ High-dose pelvic radiotherapy is a common treatment for rectal cancer. Evidence has shown that the ovaries are extremely sensitive to radiation, with doses less than 2 Gy leading to 50% depletion of immature oocytes.^21^ High-dose pelvic radiation therapy may cause acute ovarian failure and premature menopause.^22^ Especially for patients with rectal cancer who receive higher doses of radiation directly to the uterus, this treatment significantly increases adverse pregnancy outcomes and reduces the patients’ fertility.^23,24^ In male patients with rectal cancer, the testicles received 3%-17% of the dose administered.^19,25,26^ Therefore, testicular exposure to radiation in men with primary rectal cancer of all ages may lead to impaired physical, psychological, sexual function and fertility. Therefore, to fully understand the impact of CRC treatment on gonadal function in patients of childbearing age, it is crucial to analyze and vertically evaluate data related to hormone markers and reproductive health in the population of YO-CRC patients, which on the one hand, provides supportive strategies for such patients, and on the other hand, improves their fertility and further improves their quality of life.

Chemotherapy can cause the death of ovarian primitive cells, leading to premature ovarian failure and a high risk of infertility^27,28^ (Table 1). Fluorouracil has a lower impact on fertility than cyclophosphamide treatment, with a relatively lower risk of amenorrhea in women, but may cause a temporary decrease in sperm count in men.^34^ Combined treatment with oxaliplatin may also lead to transient gonadal toxicity.^30^ After treatment with oxaliplatin, the concentration of follicle-stimulating hormone in men increases, and the level of inhibin-B correspondingly decreases, resulting in a decrease in sperm count. Treatment with oxaliplatin in women can result in transient ovarian toxicity and amenorrhea, decreased anti-Mullerian hormone concentrations, and increased follicle-stimulating hormone concentrations.^30^ In a prospective study,^30^ 19 YO-CRC patients (11 females, 8 males) underwent hormone level assessments before and 6 months after oxaliplatin treatment. The results showed that the levels of anti-Mullerian hormone (estimated ovarian reserve) decreased in female patients while follicle-stimulating hormone levels increased; the level of inhibin B (estimated testicular function) in male patients slightly decreased after treatment. The results of Goossens et al^35^ showed that 66%-100% of cancer patients receiving potentially gonadotoxic treatment wanted to know the effect of the treatment on fertility. Therefore, if a patient’s treatment plan involves fertility impairment, healthcare providers should provide patients with fertility-related information, so that they can participate in making fertility-related treatment decisions.

**Table 1.** Commonly used chemotherapy drugs and related reproductive toxicity for patients with colorectal cancer.

Drugs	Toxicity of fertility
Fluorouracil	*Males*: Leading to damage of the seminiferous epithelial cells and development of vacuoles in Sertoli cells. 5FU causes significant abnormalities in spermatogenesis, including germ cell shedding, spermatid halo formation, polynucleated gianT cells, and decreased sperm count.^29^ *Females*: 5FU-induced reproductive toxicity is characterized by the presence of atretic secondary and antral follicles with reduced numbers of growing follicles, ovarian weight, and maturity impairment.^29^
Oxaliplatin	*Males*: An increase in the concentration of follicle-stimulating hormone is accompanied by a corresponding decrease in the level of inhibin B, resulting in decreased sperm.^30^ *Females*: Transient ovarian toxicity and amenorrhea, decreased anti-Müllerian hormone concentrations, and increased follicle-stimulating hormone concentrations.^30^
Capecitabine	Male mice showed degenerative changes, including decreased spermatocytes and spermatids, but the interference with oestrus was reversible. Embryotoxicity and malformation studies in mice showed a dose-related increase in fetal malformation. At high doses, abortion or stillbirth occurred in monkeys, but there was no evidence of malformation.^31^
Irinotecan	*Males*: Irinotecan administration caused acute testicular apoptosis and reduced testicular spermatogenesis, but had no effect on vascularity. Irinotecan induced long-term decreases of testicular weight, sperm count and testicular spermatogonia and caused elevated serum anti-Müllerian hormone.^32,33^ *Females*: Irinotecan administration induced acute ovarian apoptosis and decreased vascularity, as well as a mild, statistically significant, long-term decrease in the number of growing follicles, ovarian weight, and ovarian reserve.^32,33^

Abbreviation: 5FU, 5-fluorouracil.

The effect of anti-vascular endothelial growth factor monoclonal antibody inhibitors such as bevacizumab on patients’ long-term fertility was unknown. The rate of ovarian failure among patients with CRC who received bevacizumab + FOLFOX was 34%, as compared with 2% among those who did not utilize bevacizumab, and approximately 20% of women regained ovarian function at the end of bevacizumab therapy.^4,36^ Animal repeated dose toxicity studies have shown that VEGF can cause decreased ovarian maturity, reduction/loss of corpus luteum, loss of ovarian and uterine weight, and reduced number of menstrual cycles.^37,38^ Thus it may reduce female fertility. However, its impact on male fertility has not been studied.

Immunotherapy has been one of the research hotspots in the field of CRC in recent years. Studies have shown that patients with mismatch repair-deficient or microsatellite instability-high were the main beneficiaries of immunotherapy.^39,40^ Patients with partial mismatch repair-proficient or microsatellite stability CRC receiving combination therapy with other treatments such as radiotherapy and chemotherapy can respond to neoadjuvant immunotherapy.^41^ Although immunotherapy has certain effects on CRC patients, it also affect the reproductive function of young patients to a certain extent. Immune checkpoint inhibitors (ICI) have been shown to cause primary and secondary hypogonadism and, theoretically, sexual dysfunction^42^ (Table 2). However, the actual effects on gonadal function and ovarian reserve remain unknown. Animal studies^43-45^ have shown that ICI antibodies can bind to ovarian tissue, increase immune cell infiltration and tumor necrosis factor-alpha expression in the ovary, reduce ovarian follicular reserve, and impair oocyte maturation and ovulation ability; it binds to testicular tissue and impacts gametes and fertility. Impaired spermiograms and testicular histopathology have been found in men treated with ICI in different case reports and case series, and there is evidence that ICI causes primary hypogonadism or direct testicular damage.^46-48^ In few case reports published to date, no malformation or serious disease has been reported in children born to mothers treated with ICI during pregnancy.^49-52^ However, a 4-month-old infant developed immune-related enteritis after in utero exposure to pembrolizumab.^53^ In current clinical trials, the main focus was on the pathological response rate of patients, and the impact of ICI on fertility, the possibility of subsequent conception and potential pregnancy complications are important issues that need to be addressed urgently.

**Table 2.** Overview of immune or targeted drugs and effects on fertility.

Categories	Drugs	Targets of action	Effects of animal experiments on female fertility	Effects of animal experiments on male fertility
EGFR	Cetuximab Erbitux	EGFR	Not clear	Not clear
	Panitumumab Vectibix	EGFR	Yes	Yes
BRAF V600E	Encorafenib + Cetux/Pan	BRAF V600E	No	Yes
VEGF	Bevacizumab, Avastin	VEGF	Yes	Not clear
	Cyramza, Ramucirumab	VEGFR 2	Not clear	Not clear
	Regorafenib	Multiple targets	Not clear	Not clear
	Zaltrap ziv-aflibercept	VEGFA/B	Not clear	Not clear
CTLA-4 inhibitors	Ipilimumab + Nivolumab	PD-L1	Not clear	Not clear
PD-1/L1 inhibitors	Pembrolizumab + Lambrolizumab	PD-1	No	No
	Opdivo + Nivolumab	PD-1	Not clear	Not clear

Abbreviations: CTLA-4, cytotoxic T-lymphocyte antigen 4; EGFR, epidermal growth factor receptor; PD-1/L1, programmed cell death 1/ligand 1; VEGF, vascular endothelial growth factor.

## Fertility preservation methods

### Ovarian transposition

It is suitable for pelvic radiotherapy without chemotherapy or low gonadal toxicity chemotherapy. Ovarian transposition is performed to preserve ovarian function in young women who are at risk for developing premature ovarian failure after pelvic radiotherapy (Table 3). Lea Tessier et al’s^60^ systematic review and meta analysis of 10 studies showed that laparoscopic ovarian transposition may be an effective technique to reduce the radiation dose to the ovaries for premenopausal patients with anorectal malignant tumors receiving pelvic radiotherapy.

**Table 3.** Benefits, drawbacks, and available data for different fertility preservation techniques in young-onset colorectal cancer.

Method	Description	Benefits	Drawbacks	Outcome data
Ovarian transposition	Removing the ovaries from the radiation field in patients undergoing pelvic or abdominal radiation therapy	Protecting the ovaries from the toxic effects of radiation therapy	For patients who require in vitro fertilization, it will affect future transvaginal egg retrieval; limited treatment for patients over 40 years old; not suitable for patients undergoing whole-body radiotherapy	Successful delivery after ovarian transposition was rare
Uterine transposition and fixation	The uterus was repositioned into the upper abdomen to avoid radiation exposure and then repositioned into the pelvis after treatment	Protecting the uterus from the toxic effects of radiation therapy	It was experimental, and no data on subsequent pregnancy outcomes were available	Data on pregnancy outcomes were lacking
Embryo cryopreservation	The follicle-stimulating hormone was injected daily from the beginning of the menstrual period for approximately 10-14 days, and then oocytes were retrieved by transvaginal ultrasound-guided puncture. The retrieved oocytes were fertilized in vitro and finally cryopreserved	Embryo cryopreservation is the preferred method of fertility preservation for adult married women and currently has the highest pregnancy rates	The need for a male partner or sperm donor; the time required for ovarian stimulation, preparation before the in vitro fertilization cycle, and initiation and completion of the cycle may delay cancer treatment	With vitrification, the survival rate of embryos recovered was more than 95%, the cumulative pregnancy rate was 66%, and the live birth rate per patient was 44%^54^
Oocyte cryopreservation	Ovaries were stimulated and oocytes were retrieved under ultrasound guidance	No male partner is needed	Artificial reproductive technology is required for becoming pregnant. The utilization rate of oocytes for live birth is directly affected by the age of the woman, and it is recommended that the age is not more than 35 years old	The survival rate of oocytes after vitrification was 80%-95%, the average clinical pregnancy rate of embryo transfer cycle was 30% (10%-59%), and the cumulative live birth rate was 33% (6%-62%)^55,56^
Ovarian tissue cryopreservation	The ovarian tissue was removed prior to radiotherapy and reimplanted at the end of cancer treatment	It can be done at any time without ovarian stimulation and usually does not delay tumor treatment; applicable to female patients of any age; it can be combined with embryo or mature oocyte freezing technology to increase the probability of successful fertility preservation	The age limit was 35 years; in the process of reimplantation of ovarian tissue, the remaining malignant tumor cells in the ovarian tissue may be implanted into the body at the same time, leading to tumor recurrence	The pregnancy rate of ovarian tissue transplantation was 23%-31%^57^
In vitro maturation	Removing the immature cumulus-oocyte complex from antral follicles and promoting its development and maturation in vitro	The oocytes can be retrieved while avoiding ovarian hyperstimulation; only a short period of ovarian stimulation before anti-cancer treatment, or even no ovarian stimulation will not delay the patient’s anti-cancer treatment	Only be performed when oocyte cryopreservation is required but ovarian stimulation is not feasible	The IVM rate was 60%-75%, the clinical pregnancy rate was 36.1%, and the live birth rate was 25.9%^58,59^
GnRH-a	GnRH-a was used 1-2 weeks before chemotherapy and 3-6 months according to the course of chemotherapy	Protecting the cytotoxicity of chemotherapy to follicles and reducing the risk of insufficient ovarian reserve caused by chemotherapy	Most studies evaluating GnRH-a as protective agent have been conducted in women with breast cancer, and no studies have evaluated the efficacy of GnRH-a in CRC patients	There was insufficient evidence for its effectiveness

Abbreviations: CRC, colorectal cancer; GnRH-a, gonadotropin-releasing hormone-agonist; IVM, in vitro maturation.

### Uterine transposition and fixation

Oophoropexy may remove the ovaries from the radiation range, but the uterus will still be irradiated, which may cause uterine volume reduction, endometrial damage, and fibrosis.^61^ Research suggests that the uterus can be fixed on the anterior abdominal wall to reduce the dose of uterine radiation.^62^ Uterine transposition, a new technique for fertility preservation, involves positioning the uterus into the upper abdomen to avoid radiation and then repositioning it into the pelvis after treatment.^61,63^ However, the pregnancy outcomes of this technique need to be further studied.

### Embryo cryopreservation

Studies have shown that the cumulative pregnancy rate of embryo cryopreservation is as high as 60%.^64,65^ It is recommended to perform embryo cryopreservation for fertility in married women who have no contraindications for in vitro fertilization and can delay tumor treatment, but embryo cryopreservation is not recommended for tumor patients who have already undergone chemotherapy or pelvic radiation therapy. Single tumor patients under the age of 40 with normal ovarian function is recommended to undergo ovulation induction combined with vitrification cryopreservation of mature oocytes.^66^

### Oocyte cryopreservation

One disadvantage of embryo cryopreservation is the need for a fertile male partner or donor sperm, and oocyte cryopreservation avoids this prerequisite. Oocyte cryopreservation is currently the preferred technique for preserving fertility.^67^ However, the ideal number of mature oocytes that need to be retrieved for vitrification is a controversial issue. Cryopreservation of 10-15 oocytes is generally recommended.^68^ Noyes et al^69^ evaluated more than 900 deliveries with oocyte cryopreservation and showed no increase in congenital anomalies in deliveries with oocyte cryopreservation compared with natural conception.

### Ovarian tissue cryopreservation

Ovarian tissue cryopreservation is the only way to protect fertility before puberty, and it is also a preservation method for patients who do not have enough time for egg/embryo cryopreservation. This technique is suitable for patients who must undergo emergency chemotherapy. Indications for ovarian tissue cryopreservation and transplantation include: Age <37 years old; normal ovarian reserve (levels of anti-Mullerian hormone > 1.1 ng/mL, and sinus follicles > 6; before radiotherapy and chemotherapy (non-ovarian malignant tumors); patients who require normal levels of sex hormones and menstruation after radiotherapy and chemotherapy, without contraindications for hormone replacement therapy; at least 3 days before pelvic radiotherapy or chemotherapy.^66^ Fabbri et al^70^ and Elizur et al^71^ reported cases of YO-CRC patients who underwent ovarian tissue cryopreservation, followed by radiotherapy and chemotherapy with gonadal toxicity. The patient resumed regular menstruation, ovarian follicles grew normally, serum hormones returned to pre-treatment levels, and this patient eventually became pregnant. Although the study showed promising outcomes, further studies on ovarian tissue cryopreservation are needed to optimize the protocol.

### In vitro maturation

In vitro maturation (IVM) is suitable for patients with urgent tumor treatment and no time for ovulation induction, which can help avoid delays in tumor treatment.^66^

### Controlled ovarian stimulation

Controlled ovarian stimulation is for mature eggs that can be used for embryo cryopreservation or egg cryopreservation. However, the following precautions should be taken when using this technique: avoiding ovarian hyperstimulation syndrome; avoiding too long ovulation induction time; and flexibly choosing ovulation induction protocols.^66^

### Gonadotropin-releasing hormone agonists

Gonadotropin-releasing hormone agonists (GnRH-a) may protect ovarian follicles from destruction during chemotherapy by inhibiting gonadotropin levels and reducing blood perfusion in the uterus and ovaries.^72^ These agents have long been used to prevent ovarian impairment but have not been clearly shown to improve fertility outcomes and remain controversial.^73-75^ The results of a previous meta analysis which included 11 randomized controlled trials showed that more women who received GnRH-a experienced spontaneous resumption of menstruation after adjuvant chemotherapy,^76^ whereas the effect of GnRH-a in other cancers was not obvious,^77^ and there was no study to evaluate its effect in YO-CRC patients. Given the lack of effective evidence on the role of GnRH-a in fertility preservation, the ASCO guidelines clearly state that GnRH-a is not currently considered a mature method for preserving fertility.^6^

### Testicular tissue cryopreservation

Cryopreservation of testicular tissue (spermatogonial stem cells) in patients with prepubertal tumors can provide the possibility of future fertility. The strategy of orthotopic, heterotopic, and allogeneic transplantation after testicular tissue resuscitation to achieve preservation and restoration of fertility is currently a hot research area. Testicular tissue cryopreservation has good application prospects, but it is still in the exploratory stage of research.^78^

## Barriers to fertility preservation

### Doctor

ASCO points out that oncologists should discuss possible treatment-related infertility with cancer patients of reproductive age (15-49 years old) defined by the World Health Organization (WHO).^79^ However, most patients reported that doctors did not inform the risks associated with fertility before treatment. A survey of Stal et al^80^ approximately 148 rectal cancer patients showed that more than half of the survivors reported that their doctors did not discuss potential treatment-related reproductive complications with them. Approximately 75% of survivors did not store sperm (males) or eggs/embryos (females) before cancer treatment. Among them, 21.1% of males and 15.8% of females stated that they were unaware of the option to retain fertility. In another study,^81^ 44% of young survivors were uncertain whether their treatment had affected their fertility, with males reporting higher levels of uncertainty than females (50% males vs 39% females). In a cross-sectional study,^82^ the results of 101 females with YO-CRC illustrated that although more than half (52.5%) of participants reported discussing reproductive health with healthcare providers, up to 43.6% did not discuss related topics. The reasons for the above may be multifaceted: (1) Medical staff lack knowledge and training on fertility in tumor patients. Ussher et al^83^ surveyed 263 healthcare professionals and found that up to 71% of respondents did not receive professional training on cancer-related reproductive knowledge. As the main source of patient education, the tumor treatment team lacks understanding of gonadal toxicity and the patient’s need to preserve fertility, which will lead to insufficient awareness among patients. The lack of knowledge and training on fertility in tumor patients makes it difficult for medical oncologists to deeply discuss fertility issues or introduce various alternative fertility preservation measures with patients; (2) there were subjective stereotypes about patients.^83^ Medical oncologists usually avoid discussing fertility issues with patients with poor prognosis, or think that single patients, patients who have given birth, and older patients have no fertility needs, and do not discuss fertility issues with them.

### Patient

High costs lead to insufficient preservation of fertility, personal survival and cancer treatment lead to neglect of fertility issues, and genetic risks lead to fear of fertility. Garg et al^84^ included treatment costs for young patients with CRC in 14 countries. Five studies focused on prevalent disease and reported annualized per-capita cost of prevalent YO-CRC, ranging from $2263 to $16 801; 9 studies evaluated the cost of newly diagnosed diseases and showed that the average per capita cost for YO-CRC patients 12 months after diagnosis ranging from $23 368 to $89 945. This huge financial burden is an important barrier to preserving fertility. The burden of disease is also a reason for insufficient preservation of fertility. Most patients are most concerned about personal survival and cancer treatment issues in the early stages of diagnosis. At this stage, their primary information needs are related to survival and treatment. They may not be aware of the potential risk of impaired fertility or worry that preserving fertility may delay treatment, and may not prioritize fertility issues.^79^ On the other hand, the worry is that the disease will be passed on to the child. One study reported that 65% of young adult female cancer survivors were concerned about passing on the risk of cancer to their children, and their fear of heredity led them to decide not to have children.^5^

### Support system

In clinical settings, time constraints are the most commonly reported barrier in family planning discussions. Research conducted in Japan has shown that the main barrier to retaining fertility counseling includes time constraints.^85^ To overcome this barrier and ensure appropriate counseling, educational materials may help discuss fertility issues. Shin et al^86^ reported that approximately 50% of young women with breast cancer were unaware of the effects of cancer treatment on ovarian function and fertility, but only 2% of patients were unaware of the related knowledge after watching an educational video. Therefore, it is necessary to advocate for the establishment of teams in oncological surgery, obstetrics and gynecology, and reproductive medicine departments to interact with patients through establishing fertility consulting groups, improve patients’ understanding of fertility preservation such as egg and embryo cryopreservation and ovarian tissue cryopreservation, and make reasonable decisions.

Cancer patients of childbearing age desire family and social support in the process of reproductive decision-making, which may be due to the psychological burden brought by unfinished reproductive tasks and disease challenges, and they hope to get support from families and society. Good family relationships and social support can alleviate patients’ great pressure. Therefore, communication between patients and their families should be strengthened to enhance their positive experiences. In addition to providing treatment and reproductive information for cancer patients of childbearing age, medical personnel should also care for patients from the perspective of humanistic care, while improving their medical experiences and eliminating their reproductive worries.

## Relationship between YO-CRC fertility and pregnancy outcomes

Pregnancy in women with YO-CRC may increase the risk of adverse pregnancy and neonatal outcomes. Data from a large case-control study^87^ help us analyze whether cesarean delivery is associated with the risk of YO-CRC. A total of 564 patients with YO-CRC and 2180 matched controls were included in the study, and the results showed that cesarean delivery (C-section) was not associated with YO-CRC compared with vaginal delivery. However, after adjusting for factors related to maternal pregnancy, the results illustrated that women undergoing C-section had a higher risk of YO-CRC. Compared with vaginal delivery,^88^ C-section has been proven to destroy the microbial colonization of newborns and may lead to later-life diseases such as diabetes and obesity in offspring,^89,90^ which are risk factors for YO-CRC. Meanwhile, childbirth of patients with CRC may bring certain risks to mothers or newborns. A retrospective cohort study^91^ from Western Australia reported significantly increased risks of prenatal/postpartum hemorrhage, cesarean section, low Apgar scores in infants, and neonatal resuscitation in women who became pregnant for the first time after CRC versus randomly selected women with no history of cancer. Another large population-based database of 306 pregnant women with CRC and 40 887 353 pregnant women without CRC showed higher incidence rates of maternal death, arrhythmia, acute kidney injury, severe sepsis, and respiratory failure in those with CRC.^92^ In addition, the incidence of cesarean section, preterm birth, and intrauterine death was higher in the CRC group. Another study^93^ also reported increased rates of preeclampsia and cesarean section in women with YO-CRC. A maternal history of YO-CRC was also associated with preterm birth. Pregnancy of women with CRC not only poses risks to newborns but also increases risks to patients themselves. The results highlight the need for close monitoring and active management of this high-risk population and underscore the importance of early detection, timely intervention, and comprehensive management of CRC during pregnancy.

## Conclusion

Patients of reproductive age with newly diagnosed CRC should be informed about the potential impact of specific treatment options on fertility. Most patients treated with radiotherapy for CRC develop ovarian failure. A baseline fertility assessment and a discussion of fertility options in future family building are recommended. Fertility preservation is available in many ways, and healthcare providers should discuss options for fertility preservation with all patients of reproductive age who are diagnosed with cancer. Many factors affect the fertility of young patients with CRC, and medical oncologists should provide personalized guidance for patients. Adverse pregnancy outcomes may occur in cancer patients. Therefore, this high-risk population should be closely monitored and actively managed to achieve early detection, timely intervention, and comprehensive management during pregnancy.

## Data Availability

No new data were generated or analyzed in support of this research.
